# Heterogeneous expression of long noncoding RNA RP11-109D20.2: Insights into regulatory gene expression roles in colon cancer

**DOI:** 10.22038/ijbms.2025.81777.17688

**Published:** 2025

**Authors:** Sara Chitgaran, Reihaneh Alsadat Mahmoudian, Seyed Saeed Khatami, Fatemeh Nasrabadi, Ehsan Soltani, Amirnader Emami Razavi, Fatemeh Kamali, Ahmad Reza Bahrami, Maryam Moghaddam Matin, Moein Farshchian

**Affiliations:** 1 Department of Biology, Faculty of Science, Ferdowsi University of Mashhad, Mashhad, Iran; 2 Metabolic Syndrome Research Center, Mashhad University of Medical Sciences, Mashhad, Iran; 3 Department of Cancer Surgery, Surgical Oncology Research Center, Mashhad University of Medical Sciences, Mashhad, Iran; 4 Iran National Tumor Bank, Cancer Institute of Iran, Tehran University of Medical Sciences, Tehran, Iran; 5 Industrial Biotechnology Research Group, Institute of Biotechnology, Ferdowsi University of Mashhad, Mashhad, Iran; 6 Novel Diagnostics and Therapeutics Research Group, Institute of Biotechnology, Ferdowsi University of Mashhad, Mashhad, Iran; 7 Stem Cell and Regenerative Medicine Research Group, Academic Center for Education, Culture, and Research (ACECR), Khorasan Razavi, Mashhad, Iran

**Keywords:** Biomarker, Colorectal cancer, Gene expression profiling, Long noncoding RNA *RP11-109D20.2*, RNA sequencing analysis

## Abstract

**Objective(s):**

Colorectal cancer is one of the deadliest cancers worldwide, which can be prevented and even cured by early diagnosis and more efficient treatment modalities. Comprehensive transcriptional analysis has highlighted the importance of lncRNAs in CRC tumorigenesis. In this study, we identified co-expressed lncRNA networks based on public RNA sequencing data for biomarker prediction in CRC and then verified the best candidate experimentally.

**Materials and Methods:**

Publicly available RNA-sequencing data (BioProject PRJEB27536) of CRC samples and normal adjacent tissues were reanalyzed using the DESeq2 package in R to find differentially expressed lncRNAs. Pathway enrichment and gene network analysis were accomplished using GSEA and WGCNA to identify potential functions of lncRNAs with possible roles in tumorigenesis pathways. Subsequently, the expression of *RP11-109D20.2* (*lnc-Duox2-1:1*) was assessed in fresh/frozen tissues obtained from 46 CRC patients by quantitative RT-PCR.

**Results:**

A total of 17939 DElncRNAs were identified between CRC and normal tissues *via* bioinformatics analyses. A significant up-regulation of *RP11-109D20.2* (48%) was observed in CRC samples. Functional enrichment analysis showed that *RP11-109D20.2* was mainly related to pathways like phosphoric ester hydrolase, oxidoreductase, phosphoric diester hydrolase, and cyclic-nucleotide phosphodiester activities. Moreover, elevated expression of *DUOX2* in tumors with high levels of *RP11-109D20.2* suggests a link between these genes.

**Conclusion:**

Our data revealed that *RP11-109D20.2* may have a considerable role in CRC progression. However, further functional analyses are essential to evaluate the probable role of *RP11-109D20.2* as a potential diagnostic marker and its potential role in the dysregulation of cyclic nucleotide phosphodiesterase genes in CRC.

## Introduction

Colorectal cancer (CRC), as a heterogeneous illness, is the second leading cause of death from cancer and the third most frequent type of cancer worldwide, according to incidence and mortality statistics from GLOBOCAN 2020 (1, 2). In Iran, CRC is the fourth most common malignancy to be diagnosed in women and the third most common in males, with an increased rate among young people (3, 4). There is a disparate distribution and irregular pattern of CRC prevalence globally and in Iran, specifically Northwest, North, and certain regions of Central and West Iran are known as places with a high prevalence of CRC (5-7). The onset and progression of CRC are associated with several genetic and epigenetic abnormalities, including microsatellite instability (MSI), chromosomal instability (CIN), DNA base excision repair failure, DNA methylation, dysregulation of microRNAs (miRNAs), and histone modifications in epithelial cells (8, 9). Epigenetic changes are crucial in the onset and progression of CRC. This includes alterations in histone modification states, abnormal DNA methylation, and the dysregulation of microRNAs and noncoding RNAs. (10). The advances in the CRC “epigenome” have revealed that almost all the CRC samples have altered expression of noncoding RNAs, which commonly controls the expression of genes and microRNAs involved in tumorigenesis. These noncoding RNAs are also employed as clinical biomarkers for diagnostic, prognostic, therapeutic, preventative, and predictive purposes (10, 11). 

LncRNAs, a family of noncoding RNAs, are well-known for their ability to regulate various biological processes in CRC. These processes include the cell cycle, proliferation, differentiation, apoptosis, DNA damage, drug resistance, epithelial-mesenchymal transition (EMT), cell migration, invasion, and metastasis (12, 13). LncRNAs are essential for many regulatory processes, such as chromatin remodeling and maintenance, DNA methylation, transcriptional/translational activation, genome imprinting, RNA decoy, dosage compensation, and tumorigenesis. They also compete with endogenous miRNAs by modulating their translational efficacy (10, 14, 15). Further studies are necessary to better comprehend the mechanisms of lncRNAs and their role in carcinogenesis, as many functions of lncRNAs in different malignancies remain unknown. Their role in tumorigenesis has been revealed through the assessment of lncRNA signaling pathways, investigation of the expression profiles of mRNAs and lncRNAs connected to them, elucidation of the regulatory mRNA-lncRNA axis, mRNA-lncRNA concomitant expression network, and interactions between transcript modifications (16, 17). CRC has been demonstrated to correlate with dysregulation of some lncRNAs, which contribute to the incidence and progression of CRC and regulate the expression of genes important in carcinogenesis (18). Some lncRNAs, like *H19*, *MALAT*, *NEAT1*, *PTENP1*, and *HOTAIR*, have cancer- and tissue-specific expression and may have oncogenic or tumor-suppressive roles, according to next-generation sequencing (NGS) studies (19). Moreover, disruption of some signaling pathways (such as WNT/β-Catenin, KRAS, TGF-β, P53, PI3K, and AKT) *via* dysregulation of lncRNA expression can contribute to the development of CRC (20). LncRNAs play crucial roles in regulating metabolism in cancer by modulating key metabolic pathways, including glycolysis, lipid metabolism, and amino acid metabolism. They can influence the expression of metabolic enzymes and transporters, thereby facilitating cancer cell proliferation and survival under metabolic stress conditions (21, 22). LncRNAs exhibit steady expression from early to metastatic stages, according to the link between their expression level and various stages of tumor growth (23). In many cancers, alterations in gene expression regulated by lncRNAs, can lead to modification of biological processes (24). However, the function and mechanism of several unidentified lncRNAs connected to the development of CRC are still unknown. New high-throughput transcriptome profiling approaches are necessary to identify the dynamics of different noncoding RNAs during tumor initiation and progression in CRC (25). 

In this study, we aimed to find new lncRNAs related to CRC as alternative biomarkers for diagnosis and treatment of this malignancy. We examined lncRNAs that were differentially expressed (DElncRNAs) across tumoral and adjacent non-tumor tissues using bioinformatic analysis of the publicly available databases. Moreover, further downstream analysis of DElncRNAs contributes to the function prediction of DElncRNAs and enriched pathways in CRC development. To confirm RNA-sequencing data, we selected *and investigated *RP11-109D20.2 expression by quantitative RT-PCR (qRT-PCR) to show its differential expression in CRC patients. 

## Materials and Methods

### LncRNA expression profiling of CRC samples from the SRA database

FASTQ files of 62 CRC RNA-seq samples and their adjacent normal tissues were retrieved from BioProject PRJEB27536 (26). Briefly, the read quality was evaluated using FASTQC (Version: 0.11.9), and the FASTQ raw data were aligned with the human reference genome (GRCh38/hg38) using HISAT2 (Version: 2.1.0); and HTSeq-count (Version: 1.99.2) was used to quantify the gene counts with GTF (gencode. v36.long_noncoding_RNAs). The DESeq2 package (27) (Version: 1.30.1) in the R suite (Version: 4.0.3) was used to identify DElncRNAs between CRC and adjacent normal tissues. 

### Functional annotation

Weighted gene co-expression network analysis (WGCNA) package (28) (Version: 1.70-3) in the R program was used to display the weighted correlation network between co-expression of DElncRNAs and DEmRNAs, thereby helping to identify the probable function of lncRNAs. The functional analysis and co-expression network visualization was carried out by Cytoscape software version 3.8.2 (http://www.cytoscape.org). A *P*-value<0.05 and |log2 Fold Change|>1 were deemed substantial for DElncRNAs (29, 30). KEGG pathways and gene ontology (GO) enrichment analyses were carried out using the ClusterProfiler in R program (Version: 3.18.1) and Org.Hs.eg.DB package (Version: 3.12.0). The vast majority of DElncRNAs or DEmRNAs and their strong link with cellular components, molecular functions, and biological processes were shown by GSEA (31). A *P*-value of less than 0.05 was used to describe the outcomes. 

### Patients and tissue samples

*The fresh and frozen tumors, along with the adjacent non-cancerous samples, were taken* *from 46 CRC patients without a history of cancer who had never received chemotherapy and radiation therapy prior to the surgery. Twenty-three samples were taken from patients with CRC at Ghaem and Omid Oncology Hospitals affiliated with Mashhad University of Medical Sciences. The Iran National Tumor Bank, established by the Cancer Institute of Tehran University of Medical Sciences for Cancer Research, kindly provided the remaining 23 samples. All tissues were collected in RNA Protect Tissue Reagent (Qiagen, Germany) after histopathological validation and kept at -20 ℃ until RNA extraction and further experiments (*32*). The Union International Cancer TNM classification was used to evaluate the patients’ clinicopathological and tumor sample data. *Table 1* displays *the patients’ clinicopathological *information, which includes age, gender, tumor size, invasion depth, histological grade, metastatic status, and TNM staging. *

### Ethics statement

*Every patient signed the informed consent form, and the study was conducted in accordance with the Helsinki Declaration. The research protocol was authorized by the ethics committee at Ferdowsi University of Mashhad (ethical code: IR.UM.REC.1400.058), Mashhad, Iran.*

### RNA extraction, cDNA synthesis, and comparative real-time PCR analysis

*As previously described (* 15 *), total RNAs were isolated from tumor and adjacent non-cancerous tissues by the* *Column RNA isolation kit (DENAzist, Iran), and their quantity and integrity were investigated using a NanoDrop spectrophotometer (WPA, Biowave II*^+^*, Germany) and electrophoresis on 1% agarose gel, respectively. After treating total RNA with DNase I (Thermo Fisher Scientific, Germany) to remove DNA contamination, the AddScript cDNA synthesis kit (AddBio, South Korea) was used to synthesize first-strand cDNA. *Every action was taken in compliance with the guidelines provided by the manufacturers. *The expression levels of *RP11-109D20.2* (exonic) and *RP11-109D20.2* (intronic) were assessed through comparative relative real-time PCR with SYBR Green Master Mix (AMPLIQON, Denmark) on a CFX96 BioRad System thermocycler with specific primers (*Table 2*) by calculating the relative threshold cycle values of RNA expression levels through the 2*^-ΔΔCt^* method. Beta-2-microglobulin (*B2M*) was used to normalize the data as an endogenous control for both tumor and adjacent non-cancerous (reference sample) tissues, and all experiments were accomplished in duplicate for each specimen (*33*). It was determined that *RP11-109D20.2* was up-regulated in tumor samples compared to their non-cancerous counterparts*. 

### Statistical analysis

*SPSS 26 (La Jolla, CA, USA) and GraphPad Prism 5.0 (La Jolla, CA, USA) statistical softwares were utilized to analyze and visualize the data. The study employed independent-samples *t*-test, paired samples student *t*-test, and ANOVA (Analysis of Variance) to evaluate potential associations between differentially expressed genes and clinicopathological features in 46 patients with CRC. *P-*values<0.05 were regarded as significant. *

## Results

### Transcriptome profiling

*Given the high incidence of CRC and the need for early diagnosis, we evaluated the expression levels of many lncRNAs in tumor samples relative to non-cancerous tissues, followed by assessing the expression of lncRNA *RP11-109D20.2* in patients diagnosed with CRC. *

**Figure 1:**
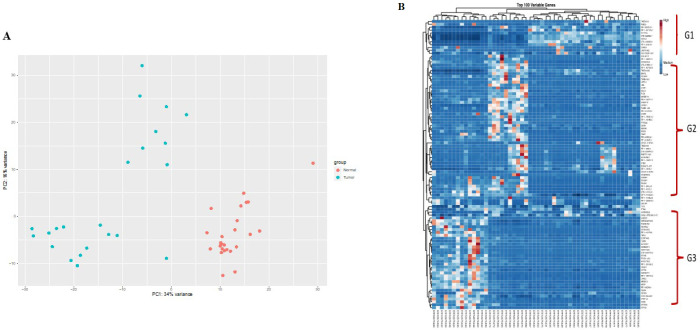
Data preprocessing and quality control of the differential gene expression analysis

*The outlier samples were found using principal component analysis (PCA). Following the exclusion of the 10 outliers, the remaining 52 samples were clustered based on gene expression patterns (* Figure 1A *). The heatmap was plotted based on the 100 variable genes in two different conditions of high- expressing *RP11-109D20.2* group versus control, which can be categorized into three different groups (G1, G2, and G3) according to the gene expression signature (*Figure 1B*).*

*The dataset contained 17939 lncRNAs (adjusted *P*-value<0.05; |log2 Fold change (log2 FC)|>1), including 695 up-regulated and 837 down-regulated lncRNAs, and the reliability of the general distribution of lncRNAs was confirmed through a volcano plot. *DOUXA1* and *DOUXA2* are among the most significantly differentially expressed lncRNAs exhibiting a similar gene expression pattern as Gene set enrichment analysis (GSEA) was used to explore the enriched biological pathways in the dataset. *Differentially Expressed Genes (DEGs) in CRC versus non-cancerous specimens. DEGs in CRC samples were implicated in many biological processes (BP), according to GO enrichment analysis, including phosphoric ester hydrolase activity, oxidoreductase activity, phosphoric diester hydrolase activity, and cyclic-nucleotide phosphodiester activity (Figure 3A). Moreover, alterations in the biological pathways and the genes involved in these pathways are indicated in a cnetplot highlighting the relationship between enriched pathways and the associated genes (Figure 3B). A large number of genes, including *PTEN*, *NT5C*, *SGPP1*, *EYA4*, *LRRC2*, *DUSP4*, and *PTPN13*, were dysregulated in the process of phosphodiester hydrolysis. Moreover, the results indicated the up-regulation of *NQO2*, *DUOX2*, and *NOS3* and the down-regulation of *AKR1C3*, *WDR93*, and *AKR1C1*. 

One important gene revealed by cnetplot is *DUOX2, *which plays a key role within the oxidoreductase activity pathway, highlighting its association with multiple other genes involved in redox-related processes and illustrating the key functional connections. The increased expression of *DUOX2*, as indicated by the color gradient, suggests its potential involvement in the oxidative stress response mechanism. Moreover, some members of the phosphodiesterase (PDE) gene family (*PDE2A/3A/4A/6A/8A/11A*) are shown to cluster around pathways related to cyclic-nucleotide phosphodiesterase activity. This clustering suggests a shared involvement of these genes in cyclic adenosine monophosphate (cAMP) and cyclic guanosine monophosphate (cGMP) metabolism, which are critical regulators of intracellular signaling.

### WGCNA identifies critical modules correlating with CRC phenotypes

*Gene networks are an effective approach to identify the correlation between genes based on their expression patterns. *23 separate gene concomitant expression modules were identified by WGCNA (Figure 4A). Moreover, the correlations between module eigengenes and phenotype of interest were indicated as a heatmap plot (Figure 4B).* Accordingly, we selected *RP11-109D20.2* which was the only upregulated novel lncRNA among the top ten genes connected to *MYH15 *(a key hub gene in CRC development) based on *WGCNA (Figure 4C). 

### Expression analysis of RP11-109D20.2 in CRC patients

*We analyzed the dysregulation level of *RP11-109D20.2* by relative comparative qRT-PCR in 46 CRC patients. *Table 1* summarizes the clinicopathological features of the patients, comprising 20 (43.5%) females and 26 (56.5%) males. The patients’ mean tumor size and age ± SD were *5.978 ± 2.8185* cm* *and* 48.09 ± 14.003* years, respectively. Most of the tumor samples were moderately differentiated (33/46, 71.3%)* with* the depth of tumor invasion T3 (28/46, 60.9%) in primary stage II of tumor progression (23/46, 50%) and without lymph node metastasis (22/46, 47.8%). Moreover, most of the tumors were found in the left section of the colon (21/46, 45.7%) and were adenocarcinoma type (41/46, 89.1%). The results of qRT-PCR are displayed in *Figure 5A* as a scatter plot based on the log2FC of the lncRNA expression levels. The minimum (-6.82 and -16.03), maximum (4.24 and 4.45), and mean ± SD (2.72 ± 0.09 and -2.95 ± 4.39) of the exonic and intronic expression levels of *RP11-109D20.2*, are demonstrated in *Figure 5A*. We used two different primer sets for exonic and intronic regions to test if this novel lncRNA goes through splicing. The expression of intronic and exonic *RP11-109D20.2* had no statistically significant difference (*P*=0.22 5); consequently, the splicing process does not occur for this lncRNA (Figure 5B). The expression of exonic *RP11-109D20.2* was observed to be increased (48%) in tumor tissues in contrast to the adjacent non-cancerous tissues. *

### Correlation between RP11-109D20.2 expression level and different pathological features in CRC

*We examined the connection between the patients’ clinicopathological features and dysregulation of *RP11-109D20.2* on the progression of CRC. There was no important association between the *RP11-109D20.2* expression and clinicopathological characteristics in CRC samples.*

## Discussion

*There are limited approaches for CRC diagnosis, and most are invasive;* therefore,* discovering innovative biomarkers for early diagnosis is needed more than ever (*34*). Transcriptome profiling and bioinformatic analyses can be used to predict the role of lncRNAs in various cellular and molecular processes, taking into account their involvement in important processes, such as cell division, epigenetic regulation, alternating splicing, regulation of gene expression following transcription, metastasis, and apoptosis (*35*). *

*In this study, the transcriptome profile of CRC patients was investigated to find important novel lncRNAs by processing the data derived from the SRA database. Gene differential expression analysis revealed the abnormal expression of lncRNAs, which is linked to CRC carcinogenesis. Next, GSEA and the construction of WGCNA were performed to predict the role of the selected lncRNA. To validate the bioinformatics findings, the expression level of the chosen lncRNA was finally examined using qRT-PCR.*

*Our first finding indicated the up-regulation of *RP11-109D20.2 *(lnc-Duox2-1:1)* *in CRC patients. More information is needed about the function of *RP11-109D20.2 *or its splicing. Based on the data obtained for the expression pattern of both exonic and intronic primers, the expression levels were not statistically different, concluding that *RP11-109D20.2* is not spliced in CRC tissue. *

*The interaction of lncRNAs with different biomolecules (such as DNA, mRNAs, proteins, or miRNAs) is associated with tumorigenesis (* 36 *). Understanding the steps of tumor growth and identifying therapeutic targets is aided by the impact of lncRNAs on the expression of upstream and downstream coding genes (* 15 *). Numerous studies have demonstrated the links between lncRNA aberrant expression, immune evasion, cellular metabolic impairment, and DNA damage (* 37 *). It has been indicated that up-regulation (such as *ATB*, *BC200*, CASC15, CCAT1, CCAT2, DMTF1v4, FAL1, FAM83H-AS1, HOTAIR, HULC, Linc01106, Linc01234, Loc441461, LincDUSP, MALAT1, MYU, *and* PCAT6) and down-regulation (such as B3GALT5-AS*,* DACOR1*, and* Loc28519) of numerous lncRNAs change the expression level of some coding genes (such as E-Cadherin*, *ZO-1*, *ZEB1*, *N-Cadherin, STAT3*, *β-Catenin*, *SNAIL*, *c-MYC*, MNT, *TGF-β1*, *BCL-2*, *P53/65*, *PCNA*, *miR-613*, *RTKN*, *Vimentin*, *miR-129-5p*, *HMGB1*, *miR-663a*, *ATR*, *E2F*, *miR-642a-5p*, *SHMT2*, *miR-211*, *miR-449b-5p*, *GLI*, *MMP-9*, *miR-34a/P53*)* in CRC which is associated with enhancing the proliferation, cell cycle, EMT, invasion, metastasis, DNA methylation as well as inhibiting the apoptosis (*13*, *38*-*43*). Consequently, expression analysis, functional mechanism, and crosstalk between lncRNAs with mRNAs and miRNAs in tumor cells can introduce their specific roles as particular or common biomarkers in diagnosing CRC patients at early stages of tumor formation, hence reducing patient mortality (*44*, *45*). *Comprehensive transcriptional analysis reveals gene networks and helps to interpret the related functions in CRC development *via* bioinformatics analysis (46, 47). *Understanding the molecular pathways of tumor formation, pathophysiology, and the identification of diagnostic indicators can be improved by analyzing the DElncRNAs between the tumor and the surrounding normal tissues. Therefore, we selected *RP11-109D20.2* on chromosome 15q on the opposite strand of the *SORD *coding gene as a new lncRNA in CRC tumorigenesis. We revealed its up-regulation for the first time in CRC patients. The role of *RP11-109D20.2 *has not been determined in CRC. Generally, *the unique spatiotemporal expression of lncRNAs implies the specific functions of these molecules* (*48*). Most lncRNAs with increased or decreased partial expression show tissue-specific expression patterns compared with coding genes, as these lncRNAs are key transcripts for acquiring specific phenotypic traits (*49*). Our results indicated that increased partial expression of *RP11-109D20.2 *probably plays a tissue-specific role in CRC cells. Moreover, *RP11-109D20.2 *expression presumably affects the cAMP signaling pathway and PDE family genes with *phosphoric ester hydrolase activity,* based on *in silico* analysis. It is noteworthy to understand the interplay between the biological function of this lncRNA and cAMP signal transduction network in the tumorigenesis of different cancer types to identify appropriate biomarkers for diagnosis, prognosis, and treatment. It has been shown that cAMP functions as an intracellular messenger and regulates several physiological and pathological processes, such as transcription, metabolism, differentiation, apoptosis, cell division, and death (*50*-*52*). *The up-regulation of cAMP response element binding (CREB) is related to tumor development, proposing its oncogenic role in tumor cells (50, 53). The cAMP pathway is activated *via* binding a primary intracellular messenger (such as hormones, drugs, and neurons) to G protein-coupled receptors, which can induce transcription by activating protein kinase A (PKA) and CREB (51, 54). Several forms of human tumors involve the abnormal cAMP-PKA-CREB signaling pathway (such as breast, colorectal, ovarian, and hypophysis) through enhancing tumor cell growth, invasion, migration, and metabolism; consequently, targeting this pathway can be a good option for cancer treatment (52, 55-58). Adenylate cyclase (ADCY) (catalyzes the conversion of ATP into cAMP) mutations affect drug efficacy in various malignancies, such as lung, esophageal, and CRC (59). 

The PDE superfamily has 11 distinct gene families (*PDE1 *to *PDE11*), which regulate the cAMP level by decomposing intracellular cAMP (54). Our data demonstrated the down-regulation of numerous *PDE* genes (including *PDE2A*/*3A*/*4A*/*6A*/*8A*/*11A*/*6B*) that are involved in the activity of phosphodiester hydrolysis and cyclic nucleotide phosphodiesterase. The down-regulation of these genes leads to decomposing cAMP and increasing the intracellular cAMP level. PDE8A* showed the highest expression decrease among all the mentioned genes and seems to have a key role in the gene network. Moreover, our results indicated the association of *RP11-109D20.2* with 357 different genes, which were most closely related to *PDE8A* and *PDE4A* genes from the PDE family. Consequently, our data may indicate that *RP11-109D20.2* has affected the cAMP signaling pathway following CRC development. Taken together, our findings suggest that dysregulation of *RP11-109D20.2* can affect the upstream genes of the cAMP pathway, or the up-regulation of *RP11-109D20.2* is indicated in CRC samples due to an increase in intracellular cAMP level. *In addition, the higher gene expression level of *DUOX2* in tumor samples with elevated levels of *RP11-109D20.2* lncRNA may suggest a potential link between these genes. As other studies have revealed the role of *DUOX2 *as a risk factor for Inflammatory Bowel Disease (IBD) and its implications for CRC progression, this lncRNA may contribute to CRC progression through regulating pathways involving immune response and chronic inflammation (60).

**Table 1:** Demographic and clinical characteristics of 46 CRC patients

**F** **actor **	**P** **atients (%)**
Age (mean ± SD)	48.09 ± 14.003 years
Tumor size (mean ± SD)‌	5.978 ± 2.8185 cm
Gender	
Male Female	26 (56.5) 20 (43.5)
Race	
Persian Non-Persian	29 (63.0) 17 (37.0)
Tumor location	
Right Left Rectum	11 (23.9) 21 (45.7) 14 (30.4)
Histology	
Adenocarcinoma Mucinous Adenocarcinoma Other	41 (89.1) 4 (8.7) 1 (2.2)
Grade	
WD MD PD Unknown	6 (13.0) 33 (71.3) 5 (10.8) 2 (4.3)
Lymphatic Invasion	
No Yes	21 (45.7) 25 (54.3)
Distance Metastasis	
M0 M1	25 (54.3) 21 (45.7)
Pathological T	
T2 T3 T4	6 (13.0) 28 (60.9) 12 (26.1)
Pathological N	
N0 N1 N2 N3	22 (47.8) 11 (23.9) 12 (26.0) 1 (2.2)
TNM staging	
I II III IV	6 (13.0) 23 (50) 13 (28.2) 4 (8.7)
Peritoneal Seeding	
No Yes	43 (93.5) 3 (6.5)
Perineural Invasion	
No Yes	29 (63.0) 17 (37.0)
Extramural Blood Vessel Invasion	
No Yes	44 (95.7) 2 (4.3)
Vascular Invasion	
No Yes	21 (45.7) 25 (54.3)

WD: Well differentiated; MD: Moderately differentiated; PD: Poorly differentiated;

N0: No regional lymph node metastasis; N1: Metastasis in 1–2 regional lymph nodes;

N2: Metastasis in 3–6 regional lymph nodes; N3: Metastasis in 7 or more regional

lymoh nodes.

**Table 2:** Primer sequences, amplicon sizes, and annealing temperatures for *RP11-109D20.2 *and *B2M *used in real-time PCR

**Amplicon size (bp)**	**Annealing T, °C**	**Primer sequence**	**Gene **
**157**	65	F: CCCCCAAGATGACCTACAACA R: AGCTGGTTCAGAAGGCCAAA	RP11- 109D20.2 (intronic)
**82**	65	F: ACGAGAGTGCAAGCAGAATGC R: GGGCTGTCTTGGACACAGAGTA	RP11-109D20.2 (exonic)
**105**	65	F: AAGATGAGTATGCCTGCCGT R: CGGCATCTTCAAACCTCCAT	*B2M*

(A) Expression analysis of PRJEB27536 dataset for differentially expressed lncRNAs (DELs). Principal component analysis (PCA) of all the samples. Normal: red label; CRC: blue

label; (B) Heatmap of gene expression signature based on the most variable genes. The X axis represents the samples, and the Y axis represents the top 100 most variable genes.

Based on the gene expression pattern, there are 3 subgroups of genes (G1, G2, and G3).

**Figure 2:**
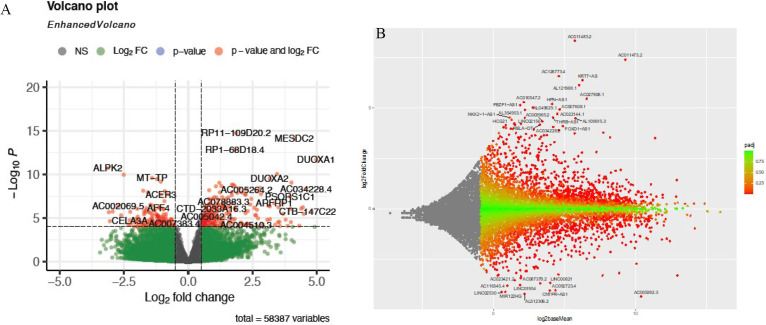
DELs between tumor and normal tissues in CRC

(A) Volcano plot of DELs between CRC and adjacent tissues in the PRJEB27536 dataset. The plots were generated using the R programming language (R 3.4.0). Statistically

significant DELs shown in red datapoints were defined with adjusted *P*-value<0.05 and absolute log2FC > 1.0 as the cut-off threshold. Different variants of *DOUX2*, including

*DOUXA1 *and *DOUXA2 *are significantly over-expressed in high vs low expressing *RP11-109D20.2 *tumor samples (B) MA plot representing log2FC between tumor and normal

samples, over the mean of the normalized counts, with red points corresponding to significantly differentially expressed genes, with an adjusted *P*-value<0.05, and without a

log2FC threshold; genes falling outside the window are plotted as open triangles. Points significantly different with FDR<0.05 are in red, and all others are in green.

**Figure 3:**
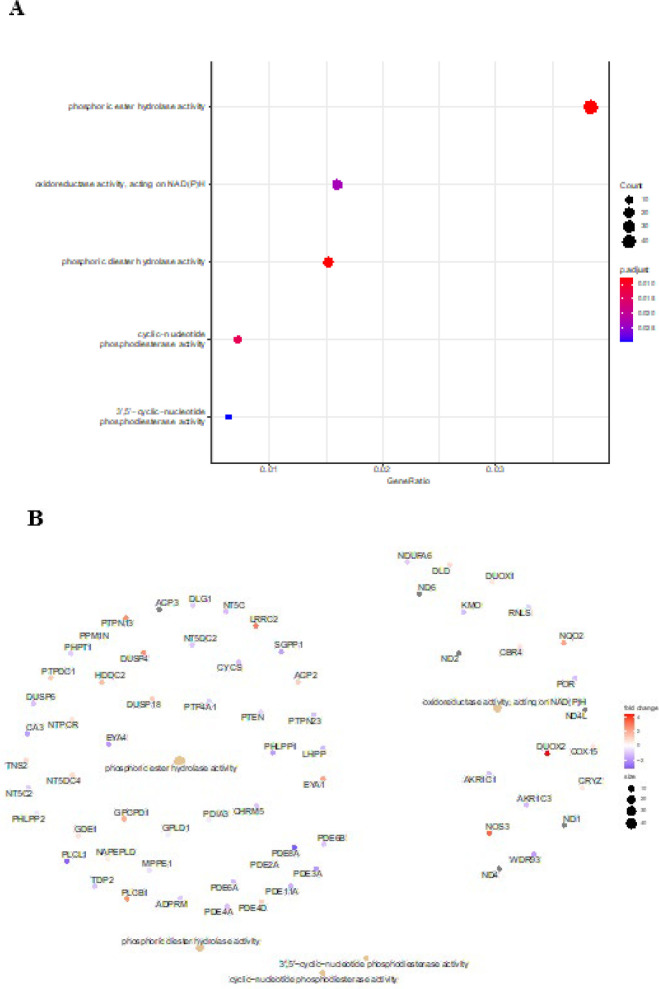
Functional enrichment and pathway analysis of differentially expressed genes (DEGs) in PRJEB27536 dataset

(A) Enriched biological pathways for all the DElncRNA; (B) Cnetplot illustrating the relationships between enriched pathways and associated genes. Nodes represent genes,

while edges connect genes to their respective pathways. The *DUOX2 *gene, prominently associated with the oxidoreductase activity pathway, stands out due to its crucial role

in redox-related processes, potentially indicating its involvement in oxidative stress response mechanisms. Similarly, members of the *PDE *(phosphodiesterase) gene family are

linked to pathways related to cyclic-nucleotide phosphodiesterase activity. The network suggests a shared function in regulating cyclic AMP and cyclic GMP signaling pathways,

highlighting their importance in cellular signal transduction. These genes' roles underscore their potential impact on the biological processes under study.

**Figure 4:**
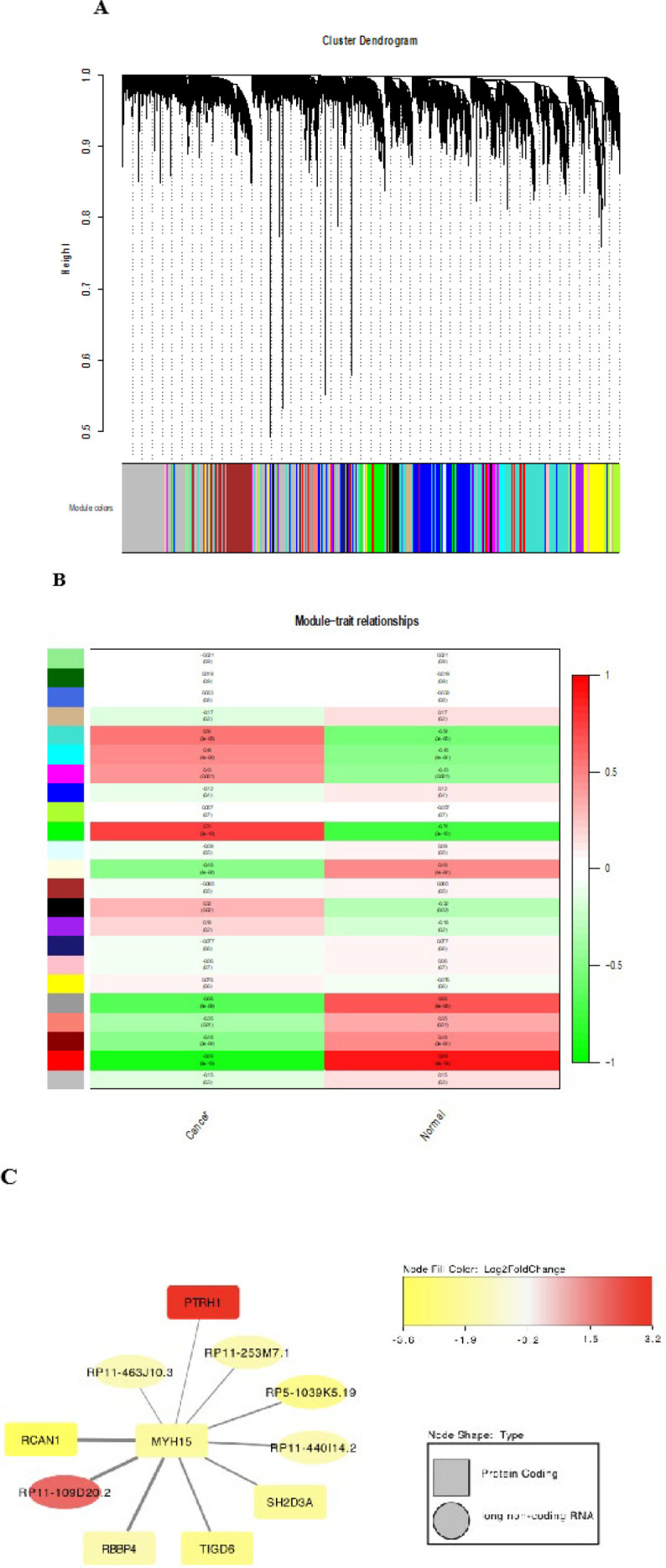
Weighted gene co-expression network analysis (WGCNA)

(A) Clustering dendrograms of differentially expressed genes in CRC. A total of 23 co-expression modules were identified. Each leaf (vertical line) in the dendrogram is assigned to one co-expressed gene, and the color in the row below the dendrogram represents the modules to which the gene belongs. The candidate lncRNA *RP11-109D20.2* was clustered in the red module; (B) Module-trait relationship plot. Each row represents module eigengenes as the representative of the specific modules. Columns correspond to the phenotype of interest (cancer). Each cell contains the value of Pearson correlation between module eigengenes and clinical traits and its corresponding *P*-value; (C) Gene network of the top 10 genes in the red module.

**Figure 5:**
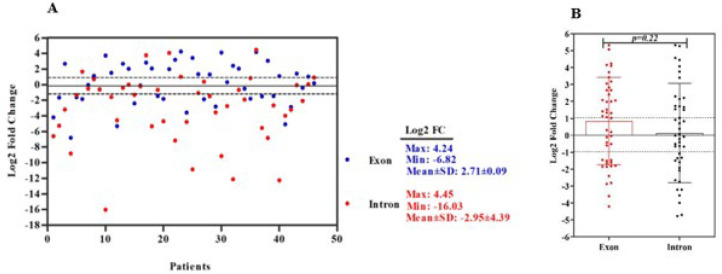
(A) Scatter plot represents a descriptive analysis of the relative expression of *RP11-109D20.2* in CRC patients. The black lines indicate the thresholds for over- and under-expression. The range between over- and under-expression shows the cases with normal expression of the lncRNA. Legends show minimum, maximum, and mean log2FC for the lncRNA expression; (B) Dot plot represents the expression of intronic and exonic* RP11-109D20.2* in CRC patients (*P*=0.225).

## Conclusion

*Our study highlighted several DElncRNAs in CRC. Here, we report the overexpression of *RP11-109D20.2 *in CRC tissue samples and its correlation to the *DOUX2* gene for the first time. However, further functional studies are necessary to assess the possible role of *RP11-109D20.2* in cancer progression.*

## Data Availability

All scripts used to analyze the data are available on Github: https://github.com/sarachitgaran/lncRNA_Biomarker_CRC.

## Declaration

Authors did not use AI tools in this manuscript.

## References

[1] Xi Y, Xu P (2021). Global colorectal cancer burden in 2020 and projections to 2040. Transl Oncol.

[2] Sung H, Ferlay J, Siegel RL, Laversanne M, Soerjomataram I, Jemal A (2021). Global cancer statistics 2020: GLOBOCAN estimates of incidence and mortality worldwide for 36 cancers in 185 countries. CA Cancer J Clin.

[3] Abbasi M, Asgari S, Pirdehghan A, Pashaki AAS, Esna-Ashari F (2021). Survival rate of colorectal cancer and its effective factors in Iran. Acta Med Iran.

[4] Arani SH, Kerachian MA (2017). Rising rates of colorectal cancer among younger Iranians: is diet to blame?. Curr Oncol.

[5] Shadmani FK, Ayubi E, Khazaei S, Sani M, Hanis SM, Khazaei S (2017). Geographic distribution of the incidence of colorectal cancer in Iran: a population-based study. Epidemiol Health.

[6] Hoseini B, Rahmatinejad Z, Goshayeshi L, Bergquist R, Golabpour A, Ghaffarzadegan K (2022). Colorectal cancer in north-eastern Iran: a retrospective, comparative study of early-onset and late-onset cases based on data from the Iranian hereditary colorectal cancer registry. BMC Cancer.

[7] Pourhoseingholi MA, Najafimehr H, Kavousi A, Pasharavesh L, Khanabadi B (2020). The spatial distribution of colorectal cancer relative risk in Iran: a nationwide spatial study. Gastroenterol Hepatol Bed Bench.

[8] Zoratto F, Rossi L, Verrico M, Papa A, Basso E, Zullo A (2014). Focus on genetic and epigenetic events of colorectal cancer pathogenesis: Implications for molecular diagnosis. Tumor Biol.

[9] Parmar S, Easwaran H (2022). Genetic and epigenetic dependencies in colorectal cancer development. Gastroenterology Rep (Oxf).

[10] Okugawa Y, Grady WM, Goel A (2015). Epigenetic alterations in colorectal cancer: emerging biomarkers. Gastroenterology.

[11] Guttman M, Rinn JL (2012). Modular regulatory principles of large non-coding RNAs. Nature.

[12] Schwarzmueller L, Bril O, Vermeulen L, Léveillé N (2020). Emerging role and therapeutic potential of lncRNAs in colorectal cancer. Cancers (Basel).

[13] Chen S, Shen X (2020). Long noncoding RNAs: Functions and mechanisms in colon cancer. Mol Cancer.

[14] Mahmoudian RA, Gharaie ML, Abbaszadegan R, Forghanifard MM, Abbaszadegan MR (2021). Interaction between LINC-ROR and stemness state in gastric cancer cells with Helicobacter pylori infection. Iranian Biomed J.

[15] Taghehchian N, Farshchian M, Mahmoudian RA, Asoodeh A, Abbaszadegan MR (2022). The expression of long non-coding RNA LINC01389, LINC00365, RP11-138J23 1, and RP11-354K4 2 in gastric cancer and their impacts on EMT. Mol Cell Probes.

[16] Iyer MK, Niknafs YS, Malik R, Singhal U, Sahu A, Hosono Y (2015). The landscape of long noncoding RNAs in the human transcriptome. Nat Genet.

[17] Chen LP, Wang H, Zhang Y, Chen QX, Lin TS, Liu ZQ (2018). Robust analysis of novel mRNA–lncRNA cross talk based on ceRNA hypothesis uncovers carcinogenic mechanism and promotes diagnostic accuracy in esophageal cancer. Cancer Manag Res.

[18] Jiang W, Xia J, Xie S, Zou R, Pan S, Wang ZW (2020). Long non-coding RNAs as a determinant of cancer drug resistance: Towards the overcoming of chemoresistance via modulation of lncRNAs. Drug Resist Updat.

[19] Aprile M, Katopodi V, Leucci E, Costa V (2020). LncRNAs in cancer: From garbage to junk. Cancers (Basel).

[20] Yang Y, Junjie P, Sanjun C, Ma Y (2017). Long non-coding RNAs in colorectal cancer: Progression and future directions. J Cancer.

[21] Wang K, Lu Y, Li H, Zhang J, Ju Y, Ouyang M (2024). Role of long non-coding RNAs in metabolic reprogramming of gastrointestinal cancer cells. Cancer Cell Int.

[22] Xu Y, Qiu M, Shen M, Dong S, Ye G, Shi X (2021). The emerging regulatory roles of long non-coding RNAs implicated in cancer metabolism. Mol Ther.

[23] Saeinasab M, Bahrami AR, González J, Marchese FP, Martinez D, Mowla SJ (2019). SNHG15 is a bifunctional MYC-regulated noncoding locus encoding a lncRNA that promotes cell proliferation, invasion and drug resistance in colorectal cancer by interacting with AIF. J Exp Clin Cancer Res.

[24] Song X, Cao G, Jing L, Lin S, Wang X, Zhang J (2014). Analysing the relationship between lnc RNA and protein-coding gene and the role of lncRNA as ceRNA in pulmonary fibrosis. J Cell Mol Med.

[25] Li M, Zhao Lm, Li Sl, Li J, Gao B, Wang FF (2018). Differentially expressed lncRNAs and mRNAs identified by NGS analysis in colorectal cancer patients. Cancer Med.

[26] Parker HR, Orjuela S, Martinho Oliveira A, Cereatti F, Sauter M, Heinrich H (2018). The proto CpG island methylator phenotype of sessile serrated adenomas/polyps. Epigenetics.

[27] Love MI, Huber W, Anders S (2014). Moderated estimation of fold change and dispersion for RNA-seq data with DESeq2. Genome Biol.

[28] Langfelder P, Horvath S (2008). WGCNA: An R package for weighted correlation network analysis. BMC Bioinformatics.

[29] Cline MS, Smoot M, Cerami E, Kuchinsky A, Landys N, Workman C (2007). Integration of biological networks and gene expression data using Cytoscape. Nat Protoc.

[30] Shannon P, Markiel A, Ozier O, Baliga NS, Wang JT, Ramage D (2003). Cytoscape: A software environment for integrated models of biomolecular interaction networks. Genome Res.

[31] Subramanian A, Tamayo P, Mootha VK, Mukherjee S, Ebert BL, Gillette MA (2005). Gene set enrichment analysis: A knowledge-based approach for interpreting genome-wide expression profiles. Proc Natl Acad Sci U S A.

[32] Ueno H, Mochizuki H, Akagi Y, Kusumi T, Yamada K, Ikegami M (2012). Optimal colorectal cancer staging criteria in TNM classification. J Clin Oncol.

[33] Nihon-Yanagi Y, Terai K, Murano T, Kawai T, Kimura S, Okazumi S (2013). β-2 microglobulin is unsuitable as an internal reference gene for the analysis of gene expression in human colorectal cancer. Biomed Rep.

[34] Bosch L, Melotte V, Mongera S, Daenen K, Coupe V, Van Turenhout ST (2019). Multitarget stool DNA test performance in an average-risk colorectal cancer screening population. Am J Gastroenterol.

[35] Guo X, Gao L, Wang Y, Chiu DK, Wang T, Deng Y (2016). Advances in long noncoding RNAs: identification, structure prediction and function annotation. Brief Funct Genomics.

[36] Yang M, Lu H, Liu J, Wu S, Kim P, Zhou X (2022). lncRNAfunc: A knowledgebase of lncRNA function in human cancer. Nucleic Acids Res.

[37] Jiang MC, Ni JJ, Cui WY, Wang BY, Zhuo W (2019). Emerging roles of lncRNA in cancer and therapeutic opportunities. Am J Cancer Res.

[38] Dilley RJ, Morrison WA (2014). Vascularisation to improve translational potential of tissue engineering systems for cardiac repair. Int J Biochem Cell Biol.

[39] Jing N, Huang T, Guo H, Yang J, Li M, Chen Z (2018). LncRNA CASC15 promotes colon cancer cell proliferation and metastasis by regulating the miR4310/LGR5/Wnt/βcatenin signaling pathway. Molecular Med Rep.

[40] Kwok ZH, Roche V, Chew XH, Fadieieva A, Tay Y (2018). A non-canonical tumor suppressive role for the long non-coding RNA MALAT1 in colon and breast cancers. Int J Cancer.

[41] Tatangelo F, Di Mauro A, Scognamiglio G, Aquino G, Lettiero A, Delrio P (2018). Posterior HOX genes and HOTAIR expression in the proximal and distal colon cancer pathogenesis. J Transl Med.

[42] Ji X, Lu Y, Tian H, Meng X, Wei M, Cho WC (2019). Chemoresistance mechanisms of breast cancer and their countermeasures. Biomed Pharmacother.

[43] Forrest ME, Saiakhova A, Beard L, Buchner DA, Scacheri PC, LaFramboise T (2018). Colon cancer-upregulated long non-coding RNA lincDUSP regulates cell cycle genes and potentiates resistance to apoptosis. Sci Rep.

[44] Cai J, Zuo X, Chen Z, Zhang Y, Wang J, Wang J (2019). Long noncoding RNAs serve as potential diagnostic biomarkers for colorectal cancer. J Cancer.

[45] Jiang C, Li X, Zhao H, Liu H (2016). Long non-coding RNAs: Potential new biomarkers for predicting tumor invasion and metastasis. Mol Cancer.

[46] Zhu M, Dang Y, Yang Z, Liu Y, Zhang L, Xu Y (2020). Comprehensive RNA sequencing in adenoma-cancer transition identified predictive biomarkers and therapeutic targets of human CRC. Mol Ther Nucleic Acids.

[47] Urh K, Zidar N, Boštjančič E (2022). Bioinformatics analysis of RNA-seq data reveals genes related to cancer stem cells in colorectal cancerogenesis. Int J Mol Sci.

[48] Ward M, McEwan C, Mills JD, Janitz M (2015). Conservation and tissue-specific transcription patterns of long noncoding RNAs. J Hum Transcriptome.

[49] Jiang C, Li Y, Zhao Z, Lu J, Chen H, Ding N (2016). Identifying and functionally characterizing tissue-specific and ubiquitously expressed human lncRNAs. Oncotarget.

[50] Sapio L, Gallo M, Illiano M, Chiosi E, Naviglio D, Spina A (2017). The natural cAMP elevating compound forskolin in cancer therapy: is it time?. J Cell Physiol.

[51] Zhang H, Kong Q, Wang J, Jiang Y, Hua H (2020). Complex roles of cAMP–PKA–CREB signaling in cancer. Exp Hematol Oncol.

[52] Zambon AC, Zhang L, Minovitsky S, Kanter JR, Prabhakar S, Salomonis N (2005). Gene expression patterns define key transcriptional events in cell-cycle regulation by cAMP and protein kinase A. Proc Natl Acad Sci U S A.

[53] Sakamoto KM, Frank DA (2009). CREB in the pathophysiology of cancer: implications for targeting transcription factors for cancer therapy. Clin Cancer Res.

[54] Serezani CH, Chung J, Ballinger MN, Moore BB, Aronoff DM, Peters-Golden M (2007). Prostaglandin E2 suppresses bacterial killing in alveolar macrophages by inhibiting NADPH oxidase. Am J Respir Cell Mol Biol.

[55] de Marval PLM, Zhang Y (2011). The RP-Mdm2-p53 pathway and tumorigenesis. Oncotarget.

[56] Wang L, Wei Z, Wu K, Dai W, Zhang C, Peng J (2018). Long noncoding RNA B3GALT5-AS1 suppresses colon cancer liver metastasis via repressing microRNA-203. Aging (Albany NY).

[57] Kwiecinska P, Ptak A, Wrobel A, Gregoraszczuk E (2012). Hydroxylated estrogens (2-OH-E2 AND 4-OH-E2) do not activate cAMP/PKA and ERK1/2 pathways activation in a breast cancer MCF-7 cell line. Endocr Regul.

[58] Peverelli E, Giardino E, Mangili F, Treppiedi D, Catalano R, Ferrante E (2018). cAMP/PKA-induced filamin A (FLNA) phosphorylation inhibits SST2 signal transduction in GH-secreting pituitary tumor cells. Cancer Lett.

[59] Zou T, Liu J, She L, Chen J, Zhu T, Yin J (2019). A perspective profile of ADCY1 in cAMP signaling with drug-resistance in lung cancer. J Cancer.

[60] Grasberger H, Magis AT, Sheng E, Conomos MP, Zhang M, Garzotto LS (2021). DUOX2 variants associate with preclinical disturbances in microbiota-immune homeostasis and increased inflammatory bowel disease risk. J Clin Invest.

